# Myopericytoma as a Differential Diagnosis of Pyogenic Granuloma

**DOI:** 10.5826/dpc.1101a105

**Published:** 2021-01-29

**Authors:** Camila Scharf, Giuseppe Argenziano

**Affiliations:** 1 Dermatology Unit, University of Campania Luigi Vanvitelli, Naples, Italy

**Keywords:** myopericytoma, pyogenic granuloma, dermoscopy, soft-tissue tumor, myofibroma

## Case Presentation

**Figure 1:**
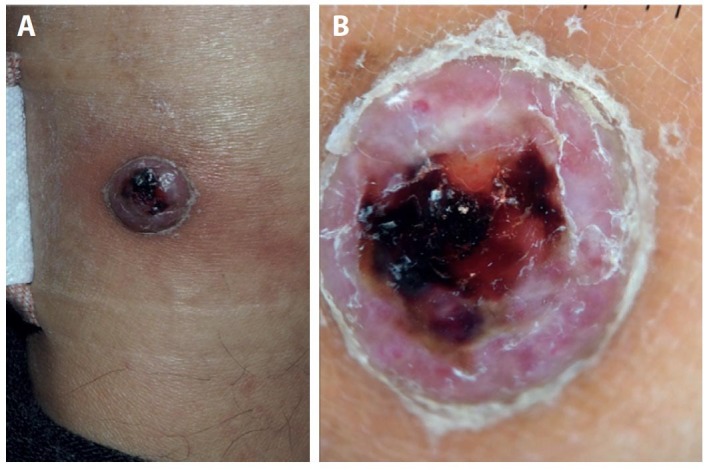
(A) Well-defined lesion, clinically consistent with pyogenic granuloma. (B) Dermoscopy showing homogeneous white-red areas surrounded by a whitish collarette.

A 46-year-old male first visited the dermatology unit complaining of a fast-growing lesion on the ankle (Figure 1A). Dermoscopy initially revealed typical findings pointing to pyogenic granuloma with homogeneous white-red areas surrounded by a whitish collarette (Figure 1B). Since the lesion was painful and friable, it was excised, and histopathology reported a myopericytoma.

## Teaching Point

Myopericytoma is a rare, benign, slow-growing soft-tissue tumor of perivascular cells. The most common location is on the distal extremities, and though the etiology is unknown, it has been associated with local trauma. Histologically, it is characterized by a well-circumscribed, non-encapsulated proliferation of spindle-shaped cells arranged in perivascular concentric rings. Therefore, it can be a differential diagnosis for hemangiopericytomas, myofibromas, and glomus tumors. Dermoscopy has been previously disclosed unfocused arborizing vessels, structureless light brown areas, and shiny white streaks [1,2]. In our case, dermoscopy presented homogeneous white-red areas surrounded by a whitish collarette and classic features of pyogenic granuloma, which was our first clinical suspicion. Dermoscopy training is essential for all practitioners and with experience we might banalize a few cases, but we must keep in mind that some dermoscopic features may be shared by different lesions.
